# Identification of Tequila with an Array of ZnO Thin Films: A Simple and Cost-Effective Method

**DOI:** 10.3390/s17122943

**Published:** 2017-12-19

**Authors:** Pedro Estanislao Acuña-Avila, Raúl Calavia, Enrique Vigueras-Santiago, Eduard Llobet

**Affiliations:** 1 Universidad Tecnológica de Zinacantepec, Av. Libramiento Universidad 106, San Bartolo el Llano, 51361 Zinacantepec, Estado de México, Mexico; pacua2@gmail.com; 2 MINOS-EMaS, Universitat Rovira i Virgili, Avda. Països Catalans, 26, 43007 Tarragona, Spain; raul.calavia@urv.cat; 3 Laboratorio de Investigación y Desarrollo de Materiales Avanzados (LIDMA), Facultad de Química, Universidad Autónoma del Estado de México, Paseo Colón Esquina Paseo Tollocan, Toluca 50200, Estado de México, Mexico; vigueras@uaemex.mx

**Keywords:** Tequila identification, ZnO thin films, gas sensor array, pattern recognition

## Abstract

An array of ZnO thin film sensors was obtained by thermal oxidation of physical vapor deposited thin Zn films. Different conditions of the thermal treatment (duration and temperature) were applied in view of obtaining ZnO sensors with different gas sensing properties. Films having undergone a long thermal treatment exhibited high responses to low ethanol concentrations, while short thermal treatments generally led to sensors with high ethanol sensitivity. The sensor array was used to distinguish among Tequilas and Agave liquor. Linear discriminant analysis and the multilayer perceptron neural network reached 100% and 86.3% success rates in the discrimination between real Tequila and Agave liquor and in the identification of Tequila brands, respectively. These results are promising for the development of an inexpensive tool offering low complexity and cost of analysis for detecting fraud in spirits.

## 1. Introduction

In the last five years, Tequila, a beverage with appellation of origin, has seen increased its exports to 182.9 million L. Meanwhile, its production has decreased by 12% and this is why the Tequila Regulatory Council [1] pursues and punishes the falsification of and fraud in this beverage. Tequila is a spirit beverage, the production of which involves different steps that include the fermentation of agave *Tequilana weber*, its distillation and, finally, its maturation in oak barrels. In contrast, other alcoholic beverages, generally designated as “Agave liquor” use cheaper alcohol sources and, instead of conducting the maturation process, mix the distilled alcohol with caramel as colorant and artificial flavors that resemble those naturally occurring in real Tequila. It is not uncommon for bottle labels employed in Agave liquors to display paintings related to agave. Even though Agave liquors are not directly dangerous to human health and are legally permitted, these low-quality spirits have often been sold as Tequila, taking advantage of their organoleptic similarities that cannot be easily perceived by an untrained drinker. This fraud results in enormous profits but clearly threatens the prestige and the market of real Tequila [2].

Fortunately, the chemical composition of real Tequila and Agave liquors is very different because of the very different nature of the products employed for obtaining these two spirits. Tequila is composed mainly of alcohol and water and comprises a powerful volatile fraction composed of 1-propanol, ethyl acetate, 2-methyl-1-propanol, 3-Metil-1-Butanol, 2-Metil-1-Butanol, 5-hidroximetyl-2-formaldehyde and 2-formaldehyde [3], among others. These species appear in mixtures and proportions that are substantially different from those found in Agave liquors, which contain artificial flavors and caramel, the latter being responsible for inducing a slightly sweet taste. Even though for a trained taster the discrimination between a real Tequila and Agave liquor is not a big challenge, trained panels are not always available and even these are subject to tiredness. In addition, buyers are not necessarily experts and this is why it is of interest to develop automated methods for rapidly determining the authenticity of a given product. Many researchers have reported sophisticated methods that include multiple steps as extraction (e.g., headspace sampling), separation, quantification, high performance liquid chromatography, isotope ratio mass spectrometry, nuclear magnetic resonance coupled with gas chromatography or electronic nose, or Fourier transform infrared spectroscopy (HPLC, IRMS, NMR GC and FTIR respectively), followed by data analysis [2,3,4,5,6]. Other molecular spectrometric analytical techniques such as UV-Vis and Raman spectrometry or chemiluminescence have been applied to tequila analysis [7]. Less frequently, fluorescence spectroscopy and photoacoustics have also been employed for discriminating fake from real Tequilas [7]. Most of these techniques are performed by highly specialized technicians employing bulky and very expensive equipment. An alternative to these instrumental analysis techniques could be the use of sensor arrays together with uncomplicated sample delivery and pattern recognition techniques. This methodology should be flexible enough to reduce substantially operation cost and obtain global information about Tequila authenticity, as it has already been reported in the analysis of other alcoholic beverages [8,9].

The analysis of beverages in general, and alcoholic beverages in particular, employing multisensory arrays and pattern recognition techniques has been the subject of intensive research in the last two decades. Two of the pioneering works consisted of discriminating between different brands of cognacs and whiskeys [10] and recognizing vineyards for red wines of the same denomination [11]. An array of semiconductor, metal oxide gas sensors was employed in these two works, which clearly showed that the choice of the sampling method implemented was very important for the success in discrimination. Since then, many authors have reported the analysis of alcoholic beverages employing multisensor systems employing metal oxides, polymers, porphyrins, phthalocyanines and hybrid gas-sensitive materials in combination mainly with resistive or gravimetric transducing schemes [8,12,13,14].

In the literature available on multisensory systems employed for beverage authentication, the sensors within the array are most of the time commercially-available metal oxides in which there is a total lack of data on the actual chemical composition, microstructure or thickness of the gas-sensitive films. As a result, little or no discussion is possible on the reasons for the usefulness (or lack of) of a given sensor within the array to discriminate among beverages or to respond to a given characteristic or target volatile fraction of the samples analyzed. In this paper, we have tested the ability of an array of very thin ZnO films to discriminate between authentic Tequila and agave liquor. ZnO was chosen as gas-sensitive material because many authors have reported its high ethanol sensitivity. For example, a recent report presented the use of randomly oriented ZnO NWs as gas sensors for ethanol [15]. Also, the sensing properties of randomly oriented ZnO over silicon substrates towards ethanol have been investigated at different temperatures and concentrations and the high response encountered was attributed to the high electron donating effect of ethanol compared to that of other gases like CO [16]. Finally, very recently we studied the implications in the nature and density of defects in vertically aligned or tilted ZnO nanowire films for the detection of ethanol or nitrogen dioxide [17]. Here, the ZnO thin films were obtained by physical vapor deposition of metallic Zn under vacuum conditions onto standard alumina transducers for resistive gas sensors. The as-deposited samples were then treated thermally under different temperature conditions and time duration with the aim of obtaining different ZnO films. These ZnO sensors integrated an array for analyzing the volatile fraction of alcoholic beverages (Agave liquor and Tequilas). The data obtained from the test were treated either by linear discriminant analysis (LDA) or the non-linear back-propagation multilayer perceptron artificial neural network (BP-MLP). 

## 2. Materials and Methods 

### 2.1. Tequila Samples

Different brands of medium and high-quality, Reposado Tequila samples were purchased from an authorized shop. These samples were coded ART, COR, 18O and DJL and their retail prices were 13, 17, 21 and 26 €/L, respectively. In addition, agave liquor at a cost of 5 €/L and coded as SB was purchased too. This agave liquor was selected because its label and lettering was similar to those employed in real Tequilas. In addition, its color was also very similar to real Tequila samples and it had been produced in the Tequila region, i.e., Jalisco. 

### 2.2. ZnO Thin Film Sensors

The sensors consisted of thin ZnO films deposited onto ceramic transducers. Zn powders from Sigma Aldrich (St. Louis, MO, USA) (99.995% pure) were used as precursor. A physical vapor deposition technique conducted under a 3 × 10^−6^ Torr vacuum and heated at 50 °C/min until first layer was deposit and stopping deposition abruptly, according to previous results, the thickness of the metallic layers was less than 20 nm. Metallic Zn layers onto the electrode area (2.5 × 2.5 mm^2^) of commercially available alumina transducers (Ceram Tech GmBH, Plochingen, Germany). Transducers comprised a pair of Pt interdigitate electrodes (front side) with an electrode gap of 300 µm, and a Pt heating resistor (back side).

Five different thermal treatments under a flow of dry air were employed to obtain different ZnO thin films. These consisted of employing three different temperatures (415, 507 or 600 °C) and different durations of the thermal treatment (15, 70 or 180 min). The resulting ZnO thin film sensors were coded according to the parameters of the treatment they had undergone, as summarized in Table 1. Samples were analyzed by scanning electron microscopy (SEM), energy dispersive X-ray spectroscopy (EDX) and X-ray diffraction (XRD).

### 2.3. Gas Sensing Tests

**Figure 1:**
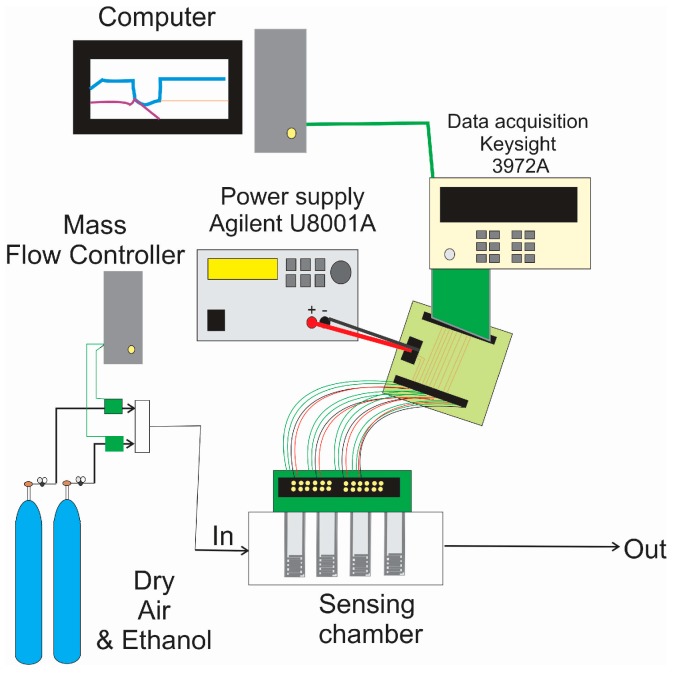
Scheme of the experimental set-up employed to study the response of the sensors towards different ethanol concentrations.

The sensor test chamber was constructed in Teflon. Its inner volume was 24 cm^3^. The chamber comprises six sockets to which sensors can be plugged in to be tested. In the first step, sensors were tested to ethanol vapors in order to study their response and to determine their optimal operating temperature. A calibrated gas bottle with ethanol in a balance of dry air, zero-grade air and a set of computer-driven mass flow controllers were employed to deliver reproducible concentrations of ethanol to the test chamber. The total flow was set to 100 mL/min. The electrical resistance of the sensors was monitored with a Keysight 3972A data acquisition system and their heating resistors were driven by an Agilent U8001A power supply. A scheme of the experimental measurement system is shown in Figure 1.

In the second step, the detection of the volatile fraction from Tequila and Agave liquor samples was considered. To help reduce the content of ethanol and water from samples that were actually delivered to the sensor chamber, a strategy inspired in the methodology described in [18] was implemented. For any given measurement, a circle of 2.5 cm diameter Whatman^®^ cellulose membrane (Merck KGaA, Darmstadt, Germany) of 1-mm pore size was spiked with 150 mL of the liquid sample and subsequently exposed to dry air for 3 min. Then, the spiked membrane was transferred to a 25-mL glass flask kept at 50 °C and dry air was used as carrier to convey the headspace of the glass flask to the sensor chamber. The flow was set again to 100 mL/min. This methodology positively weights the detection of volatiles heavier than ethanol and water, which may be representative of the organoleptic qualities of Tequila and Agave liquor samples. This sampling technique was implemented because it was judged simple to implement and inexpensive, however, other alternative techniques exist, which may have led to similar results at a higher cost. For example, the purge and trap method is a very efficient, solvent-free method for extracting the volatile fraction from solutions [19]. A careful selection of the sorbents could help reducing the amount of water and ethanol injected onto the sensor chamber [20]. Another option would be using solid-phase microextraction (SPME) in which a correct choice of the polymers coating the SPME fiber would help diminishing ethanol and water effects [21].

### 2.4. Data Analysis

The electrical resistance of the different ZnO films tested decreased when ethanol and the volatile fraction of Tequila or Agave liquor samples entered the sensing chamber. Electrical resistance increased back to its original (or baseline) value once pure dry air was flown again into de sensing chamber after a measurement was completed. The value of response (S) for any given sensor was calculated employing Equation (1):

S = (R_0_ − R_i_)/R_i_ × 100% (1)

where R_0_ was the stable value of sensor resistance in pure dry air and R_i_ the resistance value reached at the end of the measurement period (i.e., when the headspace from the sample had been delivered to the sensor chamber).

A database in the form of data matrix was gathered. Each column corresponded to the responses of a sensor and its rows corresponded to different measurements. Therefore, the data matrix had 5 columns corresponding to 5 sensors and 28 rows, which corresponded to 28 measurements performed using 4 different Tequilas and an Agave liquor. This data matrix was further analyzed by linear discriminant analysis (LDA) and by the multilayer perceptron neural network (MLP). These two pattern recognition methods were implemented from standard MATLAB (The MathWorks, Inc. Natick, MA, USA) toolboxes.

## 3. Results and Discussion

### 3.1. Morphology, Composition and Crystalline Phase of ZnO Films

**Figure 2:**
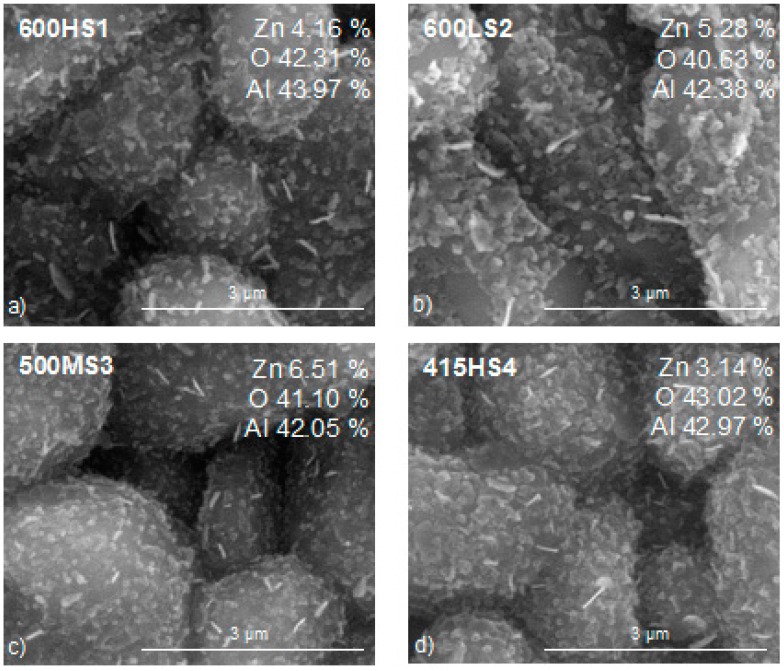

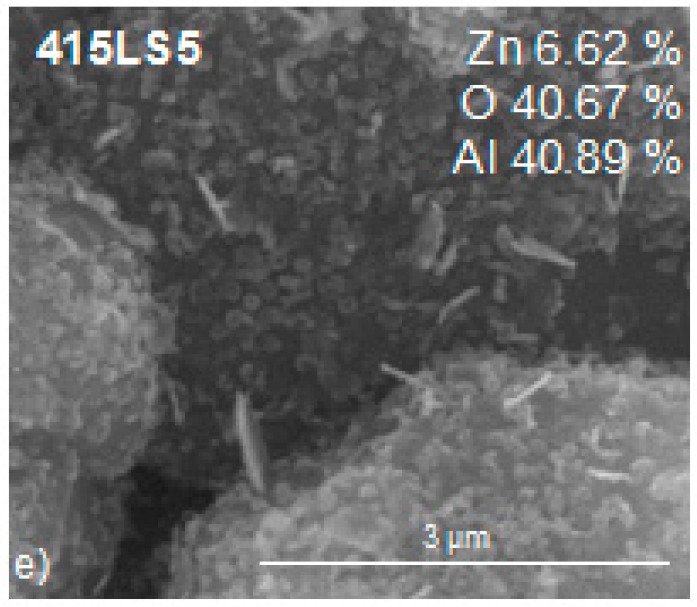
Low magnification SEM micrographs of the different thin ZnO films supported on Al_2_O_3_ substrates (big grains correspond to alumina). EDX results are indicated as insets to each micrograph. Panels (**a**–**e**) correspond to films obtained employing the five different thermal treatments implemented. Refer to Table 1 for full details.

**Figure 3:**
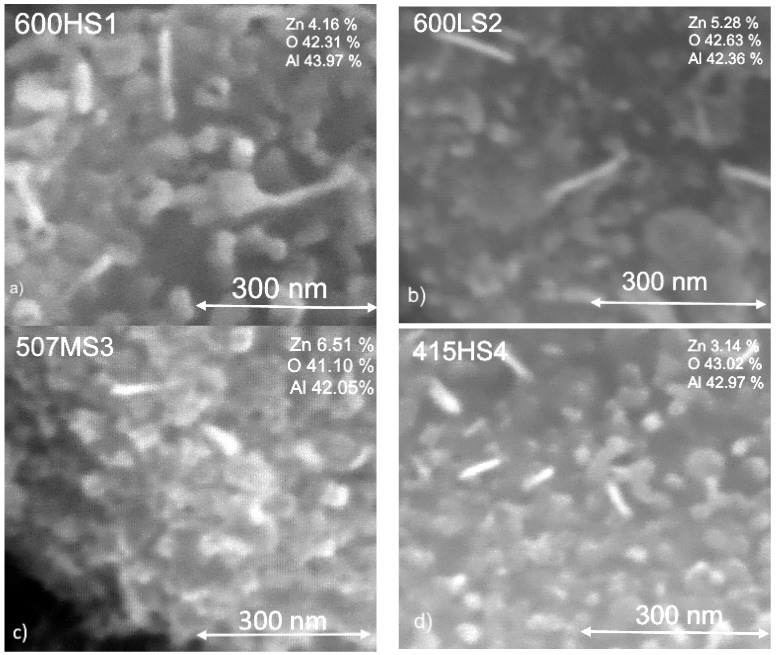

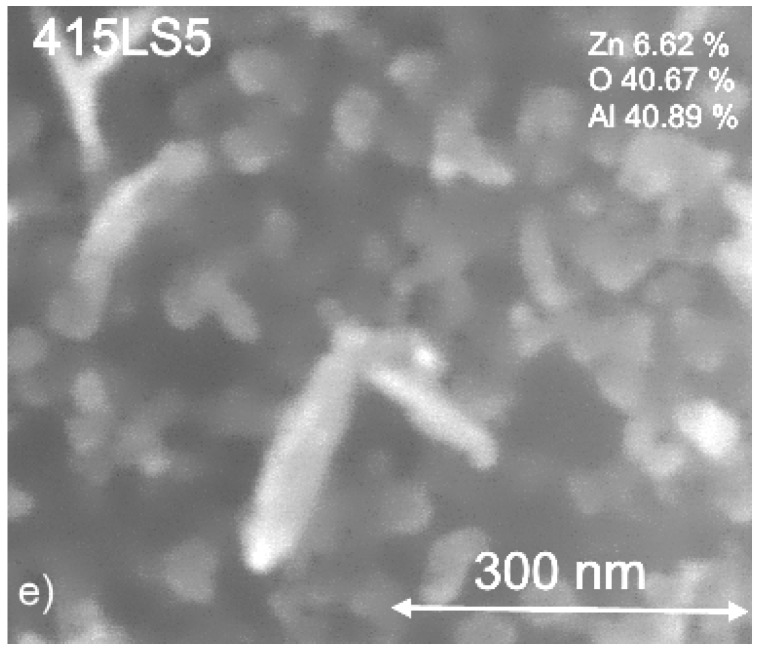
Higher magnification SEM micrographs of the different thin ZnO films supported on Al_2_O_3_ substrates. EDX results are indicated as insets to each micrograph. Panels (**a**–**e**) correspond to films obtained employing the five different thermal treatments implemented. Refer to Table 1 for full details.

The morphology and composition of the ZnO films obtained under different oxidation conditions were analyzed via SEM and EDX. Figure 2 and Figure 3 summarize these results. When the duration of the thermal treatment applied was equal or higher than 70 min, which is the case of samples 600HS1, 507MS3 and 415HS4, the morphology of the ZnO films consists mainly of pseudo-spherical nanoparticles with a few nanorods. When the duration of the thermal treatment was 10 min (case of samples 600LS2 and 415LS5), the morphology only differs slightly and the presence of some nanodisks was observed, which is indicative that some Zn structures may not be completely oxidized [22,23]. In addition, samples 600HS1, 600LS2 and 415HS4 had structures the features of which were larger than 240 nm, while samples 517MS3 and 415LS5 had in general, structures with sizes under 200 nm. In this regard, when the time of oxidation was larger than two hours or the temperature of oxidation was 600 °C, the resulting structures were larger, in good agreement to previous findings [24]. A higher duration in the thermal treatment resulted in a decrease in the room-temperature electrical conductivity of the samples, indicating an increased oxidation of the original Zn films into ZnO. EDX results indicate that the concentration of Zn was higher in 600LS2, 507MS3 and 415LS5 samples. However, the higher concentration of Zn revealed by EDX is not necessarily related to the presence of a higher amount of ZnO. In fact, the presence of nanodisks observed for samples 600LS2 and 415LS5, seems to indicate the presence of non-oxidized Zn in samples that have not undergone a long thermal treatment. These findings indicate that longer oxidation treatments at higher temperatures induce the occurrence of larger ZnO structures in nanorod and fusiform shapes, similar to those already reported in [23,25].

**Figure 4:**
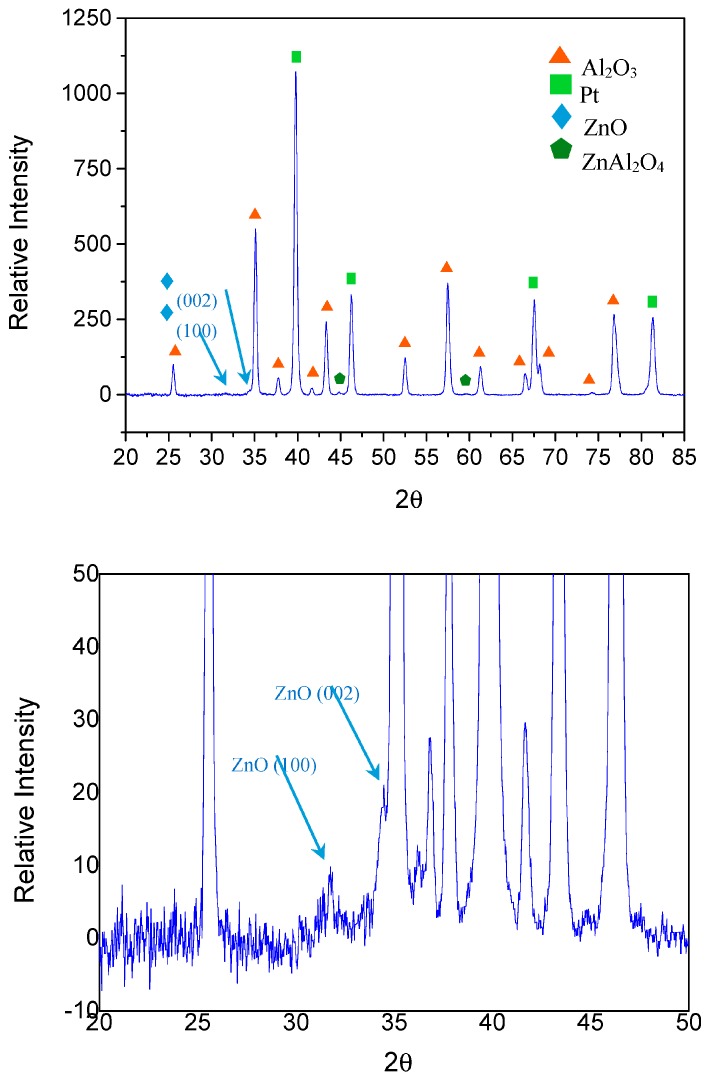
XRD analysis for sample 600HS1. The upper panel shows that the high-intensity peaks correspond to the electrodes and alumina substrate of the sensor transducer. The lower panel is an enlargement to better show peaks that correspond to hexagonal zinc oxide.

The crystalline phase of the different films was studied by XRD. All XRD results were very similar and the upper panel in Figure 4 illustrates a typical result. The highest peaks corresponded to platinum and alumina, which can be attributed to the electrodes and the ceramic support, respectively. However, a peak and a shoulder located at 31.76° and 34.39° could be attributed to the 100 and 002 planes of hexagonal ZnO, respectively (JCPDS 36-1451). In addition, two even smaller peaks located at 44.7° and 59.3° could be attributed to the 400 and 511 planes of cubic spinel-structured ZnAl_2_O_4_, respectively (JCPDS 05-0669). The low intensity of the peaks involving Zn-containing species is due to the fact that the resulting ZnO films are very thin. These results are shown in the lower panel of Figure 4.

### 3.2. Ethanol Detection with the ZnO Films

**Figure 5:**
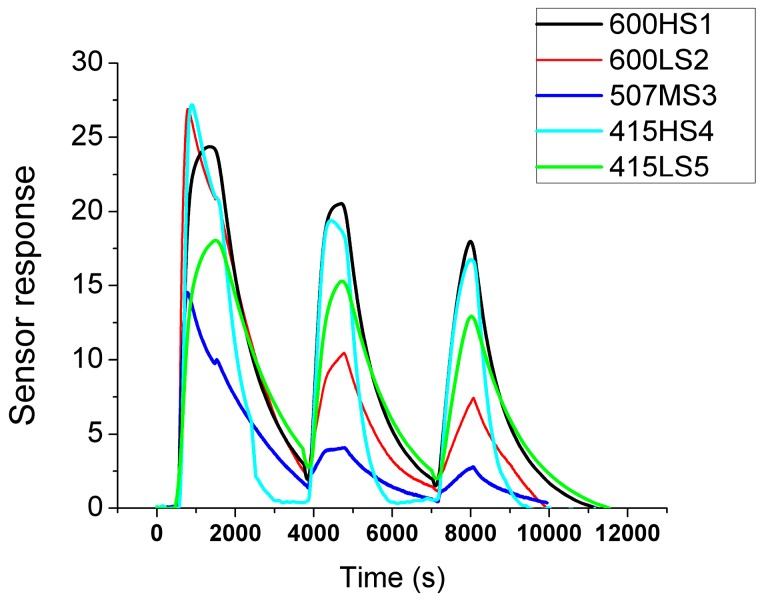
Successive response and recovery of the ZnO thin films to decreasing concentrations (20, 10 and 5 ppm) of ethanol diluted in dry air. All sensors were operated at 200 °C.

The response towards different concentrations of ethanol in air was obtained for the five different ZnO films. Figure 5 shows a typical measurement, which comprised three dynamic response and recovery cycles to 20, 10 and 5 ppm of ethanol. Response was computed according to Equation (1). Sensors were operated at 200 °C, because this temperature was found to represent a good tradeoff between response intensity and response dynamics. In particular, lower operating temperatures resulted in decreased signal to noise ratio and degraded reproducibility of results. The responses of all sensors were found to increase with increasing ethanol concentrations. There were significant differences in response intensity between the different sensors and these tended to be more evident at lower ethanol concentrations. There was no clear pattern correlating response intensity to the temperature nor the duration of the thermal treatment employed in the different films. All sensors showed similar response and recovery dynamics; however, sensor 415HS4 was significantly faster than any other sensor at recovering its baseline during the cleaning phase.

**Figure 6:**
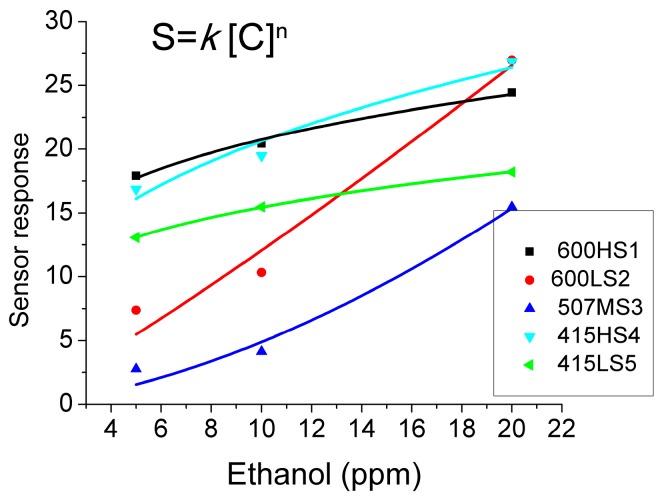
Calibration curves for ethanol and power law curves fitting for the different ZnO thin film sensors operated at 200 °C.

In a practical application of ethanol sensors like, for example, the screening of intoxicated drivers, sensors should be able to detect an ethanol concentration of approximately 200 ppm in exhaled breath, which corresponds to approximately 0.5 g of ethanol per liter of blood [26]. The results shown in Figure 5 clearly show that these ZnO films are effective for detecting ethanol at lower concentrations. Sensor response plotted as a function of ethanol concentration is shown in Figure 6. This figure shows that sensors 600HS1, 415HS4 and 415LS5 already present some evidence of response saturation at ethanol 20 ppm, which is not the case for sensors 600LS2 and 507MS3. The response, *S*, of metal oxide gas sensors is often modelled by a power law as follows:

*S* = *k*[*C*]*^n^* (2)

where *C* corresponds to ethanol concentration, and parameters *k* and *n* are constants that are useful because they provide meaningful information for comparing the performance of different sensors. The inverse of *k* (*k*^−1^) is called the response threshold. The higher *k* is, the lower the concentration of ethanol that can be reliably detected. The exponent *n* can be understood as the ability of the sensor to distinguish between similar ethanol concentrations [27] (i.e., the higher this exponent is, the higher ethanol sensitivity is).

Table 2 shows the fitting parameters for the data presented in Figure 6. Sensors 600LS2 and 507MS3 had the higher values for *n* and the lower *k* values. This is indicative that these films can distinguish between similar concentrations of ethanol (they show the higher ethanol sensitivity) and, that their response to ethanol five parts per million is rather low (their lower detection limit for ethanol is close to five parts per million). On the other hand, sensors 600HS1, 415HS4 and 415LS5 show higher *k* values, which implies that they can detect significantly lower ethanol concentrations than the minimum tested (i.e., five parts per million). However, these sensors exhibit low *n* values, which is indicative of significantly lower ethanol sensitivity. Finding a clear pattern that fully explains the behavior of the different films is not straightforward. However, according to [28], longer durations or higher temperatures of the thermal treatment generally result in metal oxides with better crystallinity and lower amount of structural imperfections than those found in samples having undergone shorter or lower-temperature treatments. Better crystallinity in ZnO films leads to a lower number of free charge carriers and higher baseline resistance, which is beneficial for detecting small concentrations of a target gas. However, structural imperfections present in metal oxides generate active adsorption sites, and these are responsible for achieving higher sensitivity over a wider concentration range of the target species.

### 3.3. Tequila Identification

**Figure 7:**
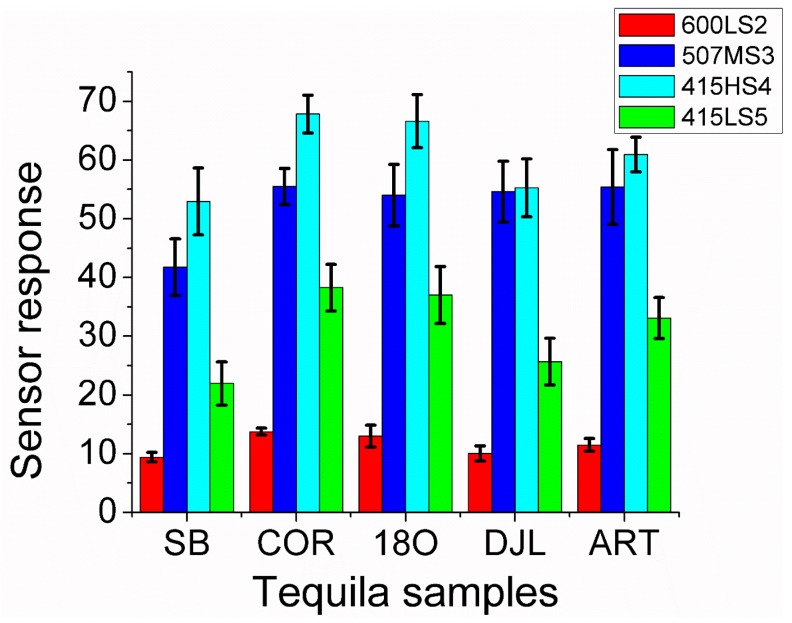
Average responses of the ZnO sensors for the different Tequila and Agave liquor samples. Sensor operating temperature was set to 200 °C. Beverage samples are labeled according to Section 2.

The different Tequila and Agave liquor samples were measured with the five-element sensor array employing the methodology described in Section 2. The responses of sensor 600HS1 and 415HS4 were almost identical and, therefore, sensor 600HS1 was no longer used in the data analysis procedures. Figure 7 shows the response of the 4 sensors retained in the array towards the different Tequila and Agave liquor samples studied. A bar in this plot shows the response of a given sensor to a type of Tequila or Agave liquor, averaged over the replicate measurements performed. Errors associated to response bars correspond to the variance over replicate measurements. Figure 7 shows how the responses of the four sensors in the array are significantly lower for Agave liquor samples (labeled SB) than for samples that correspond to Tequila. In particular, sensor 507MS3 shows a very similar response for all Tequila samples and a significantly lower response for Agave liquor. All this is indicative that there should be possible to discriminate Agave liquor from Tequila employing this sensor array.

Figure 7 also shows that the responses of sensors 415HS4 and 415LS5 present the highest differences among the samples analyzed. These two sensors were characterized by their low sensitivity to ethanol vapors (low values of parameter *n*, see Table 2), which is indicative that the differences in response arise, mainly, due to differences in the complex mixture of volatiles present in the headspace of Tequila and Agave liquor samples. Furthermore, sensors 600LS2 and 507MS3, which are the most sensitive to ethanol, show a significantly low response variability among the beverages analyzed. Therefore, small changes in ethanol concentration among the samples may have an impact on sensor response, however, the variability in sensor response observed cannot be attributed exclusively to ethanol but also to other volatile compounds, which are responsible for the differences in the organoleptic properties of the beverages studied. Previous works have faced the problems of employing metal oxide gas sensor arrays while attempting to discriminate alcoholic beverages in which small differences in ethanol concentration existed. For example, in [29] a four-element sensor array employing tungsten oxides could distinguish among methanol, ethanol and wines, but rosé and white wines were overlapped. In [9] an array of 18 commercially-available metal oxide gas sensors was employed to distinguish among beers and spirits, but it was impossible to correctly discriminate Tequilas from Whiskeys.

**Figure 8:**
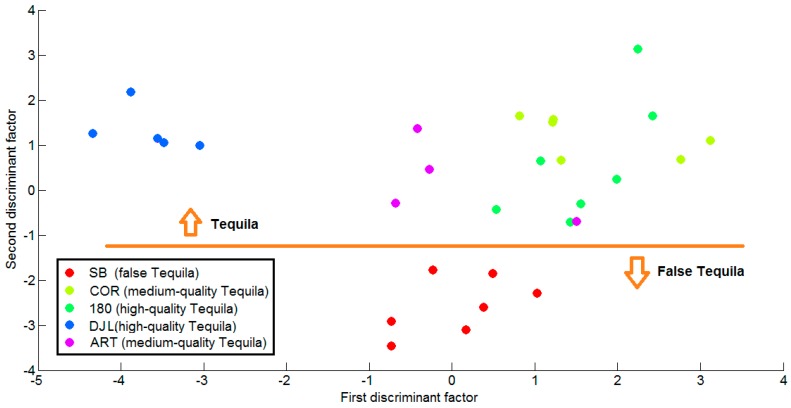
Cross-validation results of a linear discriminant analysis performed on the Tequila and Agave liquor (false Tequila) database. The first two discriminant factors account for about 97% of data variance. The orange line has been added to better show that the second discriminant factor is essential for correctly discriminating real Tequilas from Agave liquor.

A discriminant linear analysis was employed to assess whether the ZnO sensor array could discriminate among the different Tequilas. A leave-one-out cross-validation method was implemented and the plot in Figure 8 shows these cross-validation results. Given the fact that the sensors within the array are similar in nature, a mean-centering technique was employed to pre-process data. The procedure was as follows. During each iteration step of the leave-one out approach, mean centering was applied to the training subset (comprising 28 response vectors) and then a linear discriminant model was built. The vector that had been left out was then scaled employing the means computed from the training subset and subsequently projected onto the linear discriminant model. This procedure was repeated 29 times (i.e., for the total number of measurements available), each time leaving out a different response vector. The first and second discriminant factors accounted, on average, for 79% and 18% of data variance.

Checking the results shown in Figure 8 it appears clearly that the second discriminant factor helps discriminating Tequila from Agave liquor (i.e., false Tequila). While high values of the second discriminant factor are associated to real Tequilas, low values correspond to Agave liquor. A horizontal line has been added arbitrarily to the plot only to help the eye visualize the discrimination between real Tequila and Agave liquor samples.

Figure 8 also indicates that linear discriminant analysis is not able to correctly discriminate among the different real Tequila brands, since an obvious overlapping exists between COR, 180 and ART samples. The case of DJL is special because it appears clearly discriminated from the other real Tequilas. This can be explained by the fact that all Tequila and Agave liquor samples were aliquoted from freshly opened bottles, with an exception made of DJL samples. DJL aliquots were obtained from a bottle that had been opened some six months before measurements were performed and, the content of ethanol (and possibly other volatiles) in DJL samples was lower than that of all other samples. Therefore, the first discriminant factor seems to group these beverages according to their ethanol content. In other words, the first discriminant factor correlates well with ethanol concentration in the samples measured, which explains why all freshly-opened Tequilas and Agave liquor samples (showing no significant differences in ethanol content) lie in the same area. In contrast, DJL samples, which have a significantly lower ethanol concentration, show significantly lower values for the first discriminant factor.

Considering that metal oxide gas sensors in general and ZnO in particular show very good responsiveness to ethanol vapors, it is not surprising that the first discriminant factor separates samples based mainly on their difference in ethanol content. What is remarkable here is that the second discriminant factor is able to capture the subtle differences that exist between Tequilas and Agave liquor (i.e., between real and false Tequila) enabling their discrimination. Furthermore, this discrimination is robust and independent of the actual ethanol concentration in the samples analyzed, as proven by the fact that DJL samples are correctly identified as real Tequila in spite of their abnormally lower ethanol content.

To further confirm these results and check whether or not a non-linear pattern recognition method could help improving the Tequila identification power of the sensor array, the multi-layer perceptron neural network was employed. 

In the first step, the network consisted of four inputs (i.e., the number of sensors within the array), a variable number of hidden nodes and one output. This network was aimed at discriminating real Tequila from Agave liquor samples. A 100% success rate in discrimination was reached employing two hidden nodes and sigmoidal transfer functions. Once more, mean centering of data and leave-one-out cross validation were employed.

In the second step, the discrimination between samples corresponding to different Tequila brands and Agave liquor was envisaged. The network had four input nodes, a variable number of hidden nodes and five outputs (since a one of five code was employed to label the different samples). Mean centering and leave-one-out cross-validation were used. The best results were obtained when two hidden nodes were employed, reaching an 86.2% success rate in correct classification. Misclassification of three samples occurred between COR, 180 and ART Tequilas. These results confirm that the four-element ZnO sensor array is useful for correctly discriminating real Tequila from Agave liquor and that it shows some potential for identifying Tequila brands.

Two important aspects that have an impact on system performance are sensor reproducibility and sensor drift. Sensor reproducibility is important to ensure that system performance is preserved when a broken sensor is replaced by an equivalent one. Good device-to device reproducibility of our gas-sensitive films is achieved here, because sensor fabrication involves very simple, reproducible steps consisting of a physical deposition of Zn followed by an easily controlled oxidation step. Some long-term drift is unavoidable during the lifetime of metal oxide sensors, however, simple, semi-automated calibration techniques exist [30], which require the occasional measurement of a well-known species (ethanol could play this role), to ensure that the discrimination ability of the instrument does not degrade over time.

## 4. Conclusions

Different ZnO thin film gas sensors were obtained by thermally oxidizing thin Zn films that had been deposited onto commercially available, ceramic transducers. The duration and temperature of the thermal treatment were altered for achieving ZnO sensors with different gas sensing properties. SEM, EDX and XRD results indicate that there are only minor differences in the microstructure of the resulting films. However, films having undergone a long thermal treatment exhibited higher responses to low ethanol concentrations, and those resulting from short thermal treatments led to sensors with higher ethanol sensitivity. 

The sensor array was used to distinguish among different brands of Tequila and Agave liquor. Since it is well known that metal oxide gas sensors are very sensitive to alcohols and water vapor, a sampling method was implemented to minimize the amount of ethanol and water that was delivered to the sensor chamber during each measurement. Linear discriminant analysis was 100% accurate at discriminating Tequilas from Agave liquor. In addition, this method was found to be very robust and tolerant to small variations in the amount of ethanol present in the samples under analysis. This implies that the techniques developed show good potential for the development of an uncomplicated and inexpensive tool for the detection of fraud (i.e., Agave liquor is substantially cheaper than real Tequila).

A back-propagation trained, multilayer perceptron neural network reached an 86.3% success rate in the identification of Tequila and Agave liquor brands. Confusions occurred between similar Tequilas, indicating that this approach could be further developed for achieving a low-complexity and low cost of analysis instrument for an automated assessing of distinctive organoleptic properties in spirits.

## Figures and Tables

**Table 1:** Conditions of thermal treatment and code for ZnO thin film sensors.

Thermal Treatment	Code
600 °C for 180 min	600HS1
600 °C for 15 min	600LS2
507 °C for 70 min	507MS3
415 °C for 180 min	415HS4
415 °C for 15 min	415LS5

**Table 2:** Values of the fitting parameters (*k* and *n*) for the power-law response model defined in Equation (2). These values correspond to the experimental data presented in Figure 6.

Film	*n*	*k*
600HS1	0.23	12.28
600LS2	1.13	0.87
507MS3	1.65	0.10
415HS4	0.35	9.08
415LS5	0.24	8.91
